# Barriers and facilitators in the referral pathways to low vision services from the perspective of patients and professionals: a qualitative study

**DOI:** 10.1186/s12913-022-09003-0

**Published:** 2023-01-21

**Authors:** M. L. Stolwijk, R. M. A. van Nispen, A. J. van der Ham, E. Veenman, G. H. M. B. van Rens

**Affiliations:** 1 Amsterdam UMC location Vrije Universiteit Amsterdam, Ophthalmology, De Boelelaan 1117, Amsterdam, The Netherlands; 2 Amsterdam Public Health, Quality of Care, Amsterdam, The Netherlands

**Keywords:** Low vision services, Referral, Health services research, Visual impairment, Barriers and facilitators

## Abstract

**Background:**

Underutilization of and lack of access to low vision services (LVS) has been reported internationally. The purpose of this study was to identify barriers and facilitators in LVS referral procedures and service delivery from both the perspective of people with visual impairment and professionals from different eye care providers in the Netherlands.

**Methods:**

A qualitative study in the Netherlands was conducted. Barriers and facilitators were explored through semi structured interviews with older adults with macular degeneration, diabetic retinopathy and/or glaucoma (*n* = 14), and healthcare professionals including ophthalmologists and LVS professionals (*n* = 16). Framework analysis was used for analyzing the interviews with Atlas.ti software.

**Results:**

According to both patients and professionals, facilitators in LVS access and utilization are having motivation, self-advocacy, high participation needs and social support, as well as being negatively impacted by the impairment. Both samples found having good communication skills and informing patients about LVS as a healthcare provider to facilitate access. A long patient-provider relationship and the Dutch healthcare system were also mentioned as facilitators. Professionals additionally found long disease duration and the presence of low vision optometric services in the ophthalmic practice to promote access.

Barriers that were reported by patients and professionals are lack of motivation, self-advocacy and acceptance of the impairment in patients. In addition, having low participation needs as a patient, lack of information provision by providers and time constraints in the ophthalmic practice were mentioned as barriers. Professionals also reported lack of social support, short disease duration of patients, a short patient-provider relationship and lack of coordination of care in the ophthalmic practice to hinder access.

**Conclusions:**

Findings suggest that providers’ lack of information provision about LVS, especially to patients who are less assertive, hamper referral to LVS. Providers should have attention for patients’ LVS needs and actively inform them and their social network about LVS to facilitate access. Educating and training providers about how and when to address LVS may help to reduce barriers in the referral pathways. In addition, referral procedures may benefit from tools that make providers more aware of LVS.

## Introduction

Visual impairment (VI) is associated with difficulties in participating in daily activities [1, 2], increased risk of depression [3, 4], anxiety [4], fatigue [5], fall incidents and bone fractures [6]. Consequently, it can negatively affect the quality of life (QOL) of individuals. According to World Health Organizations (WHO) definition, visual impairment is defined as mild to severe vision impairment or blindness [7]. Globally, an estimated 590 million people [8] are currently affected by VI. Those with functional complaints are more at risk of having to deal with the adverse impact of the impairment.

People with a VI may benefit from low vision services (LVS), which are aimed at helping people to gain (back) independence, to fully participate in daily life activities and in society, thereby enhancing their quality of life (QOL) [9, 10]. These services may include, but are not limited to prescription of and training in the use of low-vision aids, training in orientation and mobility skills, support in daily life activities and guidance on acceptance or adaptation to the VI [11–13]. They are, among others, offered by social workers, optometrists, low vision optometrists, psychologists and occupational therapists. Some LVS have proven to be effective in enhancing the QOL and visual functioning of adults with severe VI [13]. Furthermore, from an international perspective, different institutions recommend referral to LVS in their clinical practice guidelines for ophthalmologists and optometrists, such as the American Optometric Association (United States) [14], the Royal College of Ophthalmologists (United Kingdom) [15], the Low Vision Academy (Italy) [16] and the Dutch Society of Ophthalmologists (Netherlands) [17]. Despite these efforts and supporting literature on the benefits of LVS, international studies report underutilization of and lack of access to LVS [18–21]. There seems to be a mismatch between the potential need and the actual uptake of these services. From the perspective of individuals with VI, healthcare costs, miscommunication with healthcare professionals and stigmatization by other people formed barriers in the access to or utilization of LVS [22]. From the perspective of eye care professionals, insufficient time in the ophthalmic practice, lack of knowledge and experience, and lack of funding for LVS devices seemed to hinder referral [23, 24]. From the patients’ view, self-advocacy, good communication with the professionals and social support by family and friends were some of the identified facilitators [22, 25]. Following referral guidelines was a facilitator identified by professionals [26].

Existing studies mainly examined either the perspective of patients or healthcare professionals [22–24, 26–31]. However, Khimani, Redmon [32] and Sarika, Venugopal [33] recently showed that investigating both point of views can be beneficial as it generates a broad perspective, which contributes to a better understanding about factors influencing the referral pathways to LVS. Furthermore, earlier studies were mainly conducted in countries with no systematic national provision or financial coverage of LVS and/or with a low income, such as the United States [25, 28, 32], Australia [30, 31], Canada [23], India [29, 33], Colombia [34] and Ghana [35]. As a consequence, insights to referral barriers and facilitators are particularly lacking from high-income countries in general with different types of healthcare and coverage systems.

This study aimed to explore barriers and facilitators in the referral pathways to LVS in the Netherlands from both the patient’s and the professional’s perspective. As LVS in the Netherlands are provided regionally, nationally and are well-funded by health insurance, to the best of our knowledge, this will be the first study in such a context. We focused on individuals aged 50 years or older which includes the working population, next to those at risk of the most common causes of irreversible VI [36].

## Methods

### Design

We conducted a qualitative exploratory study in the Netherlands, using semi-structured interviews with eye care professionals and patients with VI to examine barriers and facilitators in the referral pathways to LVS. The study is reported in accordance with the COnsolidated criteria for REporting Qualitative research (COREQ) [37].

### Study setting: low vision services in the Netherlands

In the Netherlands, LVS are provided by specialized for-profit low vision optometrists and three non-profit multidisciplinary LVS organizations, where LVS are offered by, among others, optometrists, advisory professionals, psychologists, social workers and occupational therapists. Low vision optometrists fit, prescribe and give advice about optical low vision aids (e.g. magnifiers and telescope glasses) during low vision optometric services that are mainly offered in hospitals, but also at patients’ homes or at LVS organizations. Low vision optometric services in hospitals are sometimes organized in cooperation with a LVS center. During such combined optometric services, patients also get information by advisory professionals of LVS organizations about which care they provide.

LVS organizations in the Netherlands offer the full range of supporting services, including advice and training in disability assistive products (e.g. cane, smartphone), mobility and orientation training and psychological therapy. All LVS are provided regionally and are largely funded by health insurance, with a compulsory deductible of 385 EUR per year (since 2016) [38]. Optical aids and other disability assistive products often need to be (partially) paid by patients, next to the compulsory deductible, depending on whether or not health insurers have a contract with the low vision aids supplier. For non-contracted low vision aids, health insurers remunerate the cost up to a maximum of 80%. According to the guideline of the Dutch Society of Ophthalmology [17], referral to a low vision optometrist needs to be considered when the visual functioning of patients can be (partially) improved or compensated with optical low vision aids. Referral to LVS organizations is recommended for patients with a visual acuity of > 0.50 logMAR (6/19 Snellen) and/or a visual field of < 30° around the central point of fixation and/or an evident request for assistance when therapeutic options in regular ophthalmic practice are insufficient. Officially, patients need to be referred by a medical specialist (e.g. ophthalmologists) or a low vision optometrist in consultation with an ophthalmologist for LVS utilization at the LVS organizations. In practice, patients sometimes contact LVS organizations without being referred (self-referral) and then need to request an official referral by their medical specialist before they can access LVS.

### Study population

#### Professionals

Eye care professionals from different healthcare institutions were recruited by purposive sampling [39] to reflect diversity in types of professionals and a mix of sex, age and years of work experience. Professionals included were ophthalmologists and LVS professionals. Inclusion criteria for professionals were: (a) contact with patients (50 +) with macular degeneration, glaucoma and/or diabetic retinopathy and, (b) involvement in the referral process to LVS or. sufficient knowledge about it by profession. Professionals employed for less than 6 months were excluded from participation.

Recruitment of professionals took place via a LVS organization, an academic and a non-academic hospital. Potential participants were contacted by email along with an information letter about the study, after which they were called to double check the inclusion criteria.

#### Patients

Patients were selected by purposive and convenience sampling [39] to reflect ophthalmic diagnoses that most often cause irreversible VI in people aged 50 years or older and the working population, and to reflect a mix of disease duration, sex, age, type of referral to LVS (self-referral, referral by ophthalmologist) and status of referral (did/did not follow up on referral). Patients were purposively recruited by an academic and two non-academic hospitals and the Netherlands Institute for Health Services, and through convenience sampling by two patient associations for general eye diseases and macular-related eye diseases via their websites and newsletters. Inclusion criteria that were checked by the recruiting institution and by telephone were: (a) 50 years or older; (b) diagnosis of macular degeneration, glaucoma and/or diabetic retinopathy; (c) referral to a LVS not longer than 6 months ago; (d) first time referral; (e) sufficient mastery of the Dutch language, and (f) cognitively able to participate in an interview. With respect to cognitive impairment, this was checked by the recruiting institution on the basis of clinical patient information, i.e. those with an intellectual disability or a cognitive impairment were excluded from the study. Furthermore, cognitive eligibility was examined by telephone based on how the inclusion conversation went.

An information letter and a participation form was sent to potential participants and they all received oral information by telephone before signing informed consent.

#### Sample size

On the basis of existing literature on qualitative research design [40, 41] and the researchers’ experience, we aimed to recruit a total of 30 participants, with 15 respondents for each of the two subgroups of patients and professionals. This sample size was deemed to be sufficient to hold enough information power [41] to contribute substantially to new understandings of the research topic in the Dutch LVS context. During the whole research process we regularly evaluated the adequacy of the pre-set sample size.

### Data-collection

A semi-structured one-on-one interview was scheduled for all eligible participants and conducted by MS; data were collected between September 2019 and April 2021. Interviews with professionals were conducted face-to-face at their workplace. However, because of COVID-19 pandemic government regulations between March 2020 and 2021, patients were interviewed by telephone.

An interview scheme was developed for each of the two subgroups separately and was based on findings from prior studies [22, 24–26], and on the Social Ecological Model [42, 43] (available upon request). According to a social ecological approach of health promotion, health is a function of an individual and his/her environment. It can be used to identify the factors that contribute to a certain health-problem on individual, interpersonal, organizational, community and public-policy level and was recently used by Kaldenberg [25] in a similar study.

Both interview schemes addressed participants’ personal experiences and behaviors relating to barriers and facilitators in the referral pathways. The interview started with (1) an introduction by the interviewer, explaining the study and interview structure, followed by (2) general questions about the participant, asking about (working) background, and VI and health characteristics (patients), (3) questions about the referral pathways, referral procedures, reasons for patients’ (self-)referral and (not) following up on referral, information provision, communication between patient and provider and registration at LVS, (4) general questions about the referral pathways, probing most relevant barriers and facilitators and advices for clinical practice, and (5) concluding questions with a summary and verification of the topics discussed during the interview. Interview schemes were pilot tested in advance.

The interviews took between 60 and 120 min. Patients received a gift certificate for their participation.

### Data-analysis

Interviews were audio recorded and transcribed verbatim. The transcribed interviews were summarized and were sent to participants for member checking [44]. This led to some additions and adjustments.

Transcribed interviews were analyzed with framework analysis [45–47] in ATLAS.ti 8 software. After reading all the transcripts and making notes to get familiar with the data (step 1), four interviews were coded independently and inductively by two researchers (MS and AvdH) and were classified into main themes and subthemes (step 2). After the researchers agreed on the coding, which was accomplished by face-to-face discussion, an analytical framework was developed that was purposefully linked to the Social Ecological model which was used as a basis for the interviews (step 3). All transcripts were then reviewed to apply the analytical framework and to chart the data into a framework matrix (step 4 and 5). Finally (step 6), we mapped and interpreted the data and established relationships by grouping themes around the levels of the Social Ecological Model [42].

## Results

### Response and characteristics

Sixteen professionals (female 40%) participated in this study (Table 1). The mean age was 47 years (range 30–64). Professionals were ophthalmologists and LVS professionals such as (low vision) optometrists*,* eligibility assessors/administrators, an advisory professional, a clinical physicist and a manager*.* The average working experience of the professionals was 15.5 years.

**Table 1:** Characteristics of the study population (*N* = 30)

	**n (%)**	**Mean (SD)**	**Median [range]**
**Characteristics professionals (*n* = 16)**			
Sex, female	6 (40)		
Age (in years)		47 (8.9)	46 [30–64]
Profession			
Ophthalmologist	7 (44)		
LVS professional	9 (37)		
Low vision optometrist	3 (33)		
Professional LVS organizations	6 (67)		
Optometrist	1 (17)		
Eligibility assessor/administrator	2 (33)		
Clinical physicist	1 (17)		
Advisory professional	1 (17)		
Manager	1 (17)		
Years of work experience		15.5 (10.2)	15 [0.5–35]
**Characteristics patients (*n* = 14)**			
Sex, female	10 (70)		
Age (in years)		74 (11.6)	74.5 [55–96]
Visual impairment			
Mild VI^b^	3 (21)		
Moderate VI^b^	8 (57)		
Severe VI^b^	1 (7)		
Monocular vision	1 (7)		
Reduced vision and visual field defect	1 (7)		
Eye disease^a^			
Macular degeneration	9 (64)		
Glaucoma	3 (21)		
Diabetic retinopathy	3 (21)		
Cataract	1 (7)		
Years since diagnosis		10 (13.1)	5 [0.3–44]
Years since referral		2 (1.8)	0.75 [0.2–6]

*SD* standard deviation

^a^Patients could have had more than one eye disease

^b^according to Word Health Organization (WHO) criteria

Fourteen patients participated in this study (female 70%). The mean age was 74 (range 55–96). Almost two thirds of patients had macular degeneration as the cause of vision loss, followed by glaucoma and diabetic retinopathy. Half of all patients initiated their own referral by asking their medical specialist or by contacting an LVS themselves after which a post-hoc referral was arranged. The other half was referred by indication of a medical specialist. All patients followed up on their referral and utilized LVS. On average, patients had been referred 2 years ago.

### Barriers and facilitators

Barriers and facilitators according to the Social Ecological Model are presented in Table 2. In order to give an indication about the number of described experiences, following system in reporting the findings has been used: One participant; Some − up to a quarter of the participants; Several − between a quarter and half of the participants; Half of the participants; The majority − between half and three quarters of the participants; (Almost) all of the participants.

**Table 2:** Barriers and facilitators in the referral pathways to low vision services from the perspective of professionals and patients

Level	*Barriers/Facilitators*	*Professionals*	*Patients*
*Individual Level*	Motivation	+ , −	+
	Self-advocacy	+ , −	+
	Experienced impact of the VI	+ , −	+
	Participation needs	+ , −	+
	Attitude regarding asking for help and seeking healthcare	−	
	Disease duration	+ , −	
	Lack of awareness and knowledge of LVS	−	
	Acceptance of the VI	+ , −	−
	Other individual factors (Cultural background, unawareness of the eye disease, overall health condition and other private circumstances)	−	
*Interpersonal Level*	Information provision LVS	+ , −	+ , −
	Communication skills/strategies healthcare professionals	+	+ , −
	Social support	+ , −	+
	Length of patient-provider relationship	+ , −	+
	Other sources of LVS information	+	+
*Organizational level*	Communication between health professionals	−	
	Cooperation between providers	+	
	Care coordination	−	
	Low vision optometric services	+	
	Time constraints ophthalmic practice	−	−
*Community Level*	Fear of stigma	−	
	Distance to LVS/Transportation	−	+ , −
	Education of healthcare providers of LVS	+	
*Public policy level*	Dutch healthcare system	+ , −	+
	Regional service provision	+	
	Long waiting lists	−	

( +) facilitator, barrier ( −)

### Individual level

#### Motivation

Patients’ motivation was identified as an important barrier and facilitator for LVS referral. The majority of the professionals with the authorization to refer patients (ophthalmologists, low vision optometrists in consultation with ophthalmologists) said that, in line with shared decision making, they only refer patients if they want to be referred and that a great amount of patients with whom they discuss LVS and offer a referral to do not want to because of various reasons.

“*It goes pretty fast, people know whether they want it or not. But there is a really large group that doesn't want it (…) I think half of the patients says ‘I'm feeling too good’ or ‘I don't want to’. Maybe even more than half (Ophthalmologist, male, aged 42).”*

Some patients mentioned that one of the main reasons to be referred was that they were open to it, willing to get help, curious about what LVS would bring them or trusted the advice of their ophthalmologist.

“I thought it was good, I think every little bit helps, so if [name LVS organization] can help me with this, for example with glasses or something, I think it's great (Male, aged 96, macular degeneration).”

#### Self-advocacy

Both professionals and patients mentioned self-advocacy to be an important facilitator. According to half of the professionals, patients who are able to express their experienced impairments in daily life, who actively ask for possibilities to enhance their visual functioning, or who actively ask for a referral, are more likely to be referred:

*“It also depends on the patients ability to self-advocate. (…) Yes, they are more likely to say that they want something or they are more likely to say to the doctor ‘refer me (to LVS)’ or ‘this does not work and that does not work anymore’ (Optometrist LVS, female, aged 39).”*

This was confirmed by several patients who mentioned to be referred after actively asking for referral in consultation with their ophthalmologist, sometimes after actively searching for information about LVS on the internet. In turn, not being an effective self-advocate as a patient was mentioned as a barrier by some professionals.

#### Experienced impact of the VI

The experienced impact of the VI was mentioned as a facilitator as well as a barrier in the referral pathways to LVS by participants. Almost all patients stated that the reason for being referred and wanting LVS was the impact of VI on their daily life, mostly in practical activities such as reading, driving, watching TV and biking, but also fatigue and work.

“Yeah, watching TV, especially reading the newspaper, what I always do. Sometimes when I was cooking food, I didn’t see it well. Yeah, very simple things actually (Female, aged 72, glaucoma).”

Several professionals stated that the practical implications of patients’ VI are often the reasons for patients to seek LVS and a starting point for professionals to talk about LVS during consultation. Several professionals indicated that they actively ask patients about their impairments in daily life activities or patients bring it up themselves*.* Furthermore, some professionals reported that mental wellbeing can be the reason to suggest a referral, for example in patients whose vision has suddenly deteriorated.

“*In the beginning you are quite restless, so I wanted to know what you (LVS) can do for me. That is the reason that I brought it up in a consultation with my ophthalmologist. (…) Yes it makes you a little anxious, ‘What else can I do, can I still help myself’? You feel you are becoming less independent, you have to ask for help (Female, aged 88, macular degeneration).”*

#### Participation needs

Several professionals thought that participation needs of patients are key in the patients’ motivation for referral. According to these professionals, how actively or in-actively patients participate in life and how important that is for them, determines if they are open to LVS and open to accept a referral. Some patients may not have participation needs anymore, possibly because they sometimes say they are ‘too old’, reflecting they have found resignation in the fact that they are not able to do certain things anymore.

*“While the one does not have that need and says ‘I am happy that I am still able to see the television and I walk a little bit outside and all that goes well’, someone else may want to drive their car or has to take care of someone and then says ‘I really need to be able to ride my bike’ (Ophthalmologist, male, aged 42)*.”

For some patients the wish to continue their participation in society, for example to be able to work (again), to participate in activities that are important to them or to participate socially stimulated their referral.

“*I thought ‘The fact that I will see less won’t bring me down’ (…), so I thought I need to make sure that I can keep reading and talk on the phone with people, internet, e-mail, using the mobile phone with the library on it and the reading function. So basically, to be able to do these activities, to stay active as much as possible (Male, aged 78, macular degeneration).”*

#### Attitude regarding asking for help and seeking healthcare

Several professionals stated that patients’ attitude regarding asking for help and seeking healthcare in general sometimes hamper patients’ motivation and in turn referral. They indicated that elderly patients are those who often refuse LVS, because of generationally typical opinions. Some believed that patients who were born before 1950 and experienced the war prefer not to ask for help.

*“A whole generation of people simply has learned to take care of themselves and not to ask for help, while I think the current generation is better able to do that than people who are aged 70 or older (LVS professional, male, aged 56).”*

#### Disease duration

From the professionals’ point of view, disease duration may influence the patients’ motivation for LVS and thus, referral. Some professionals stated that patients with a newly diagnosed eye disease, especially neovascular macular degeneration, often do not want to be referred to LVS right away, because they hope that the treatment (e.g. anti-VEGF injections) will improve their vision and therefore want to await the treatment effect*.* In the early stages, some patients first need to find out what it means to have a visual impairment and are already helped with simple advice.

*“ (…) Especially with recently diagnosed macular degeneration, people do not really want that yet. Because they are still in the beginning of that treatment and they want to see better again and they still have hope that it will all get better (Ophthalmologist, female, aged 43).”*

#### Lack of awareness and knowledge of LVS

Lack of awareness and knowledge of LVS was mentioned by professionals as another barrier in the referral pathways to LVS. Some professionals explained that patients are often unaware of the available services and are dependent on the healthcare professionals to inform them. Patients also do not always remember what is being said about LVS during a consultation, as they may be overwhelmed by the information given, which may lead to unawareness and, in turn, to a barrier to LVS access:

*“When you have a consultation with a patient, they remember 25% when leaving and 10% when they are at home. So a lot of (information) is lost. So you shouldn’t be surprised when people don’t remember that you’ve talked about it (LVS) (Ophthalmologist, male, aged 64).”*

In addition, several professionals believed that the reasons for some patients to not wanting LVS and refusing referral might be related to not knowing what to expect from a LVS.

#### Acceptance of the VI

Both professionals and patients indicated that acceptance of the VI can play a facilitating or hindering role in the referral process. Professionals indicated that some patients go through a complicated grieving process or have difficulties to adapt and think as vision loss as a temporary problem. Some patients stated that they have been informed about LVS before, but refused it because they felt not ready for it at that moment, because it would symbolize actually being visually impaired.

“*I walked in there once, during a walk-in consultation hour and that was very confronting for me. I was welcomed by a woman who was blind and she showed me around in a room and showed me all the low vision aids they had. (…) Yeah, then you are confronted with the fact ‘I need something, I'm welcomed by a blind woman, so it could get even worse, for me it could also go that way’ (Male, aged 60, macular degeneration and glaucoma).”*

Several professionals stated that problems with acceptance of the VI can be a reason to discuss LVS with patients.

*“Yeah, those are often the people who had acute vision loss. Because of a trauma or vascular occlusion or something like that and indeed for those people acceptance is often difficult and it takes time. Then I also often talk about LVS and that they can help with that (Ophthalmologist, female, aged 43).”*

#### Other individual factors

Some professionals specialized in diabetic retinopathy thought that cultural background within this patient group may also hamper LVS referral. It was mentioned that for some patients it is more common to fully rely on the competences of the medical doctor, and they may lack awareness of the positive impact their own actions can have on their disease outcome.

Another factor that was indicated as a barrier by several professionals was patients noticing late that they have problems with their eye sight. Especially in glaucoma, patients are sometimes unaware of their (severity of) visual field defects due to compensatory strategies and habituation to the impairment, resulting in late diagnosis by the ophthalmologist and late referral to LVS. Besides that, patients with glaucoma may still have a quite good visual acuity and ophthalmologists therefore might not think about referring.

The overall health condition of patients and/or other private circumstances were also mentioned to be a barrier in patients’ access by some professionals. Professionals mentioned that patients who have a bad overall health condition, e.g. serious illness or cognitive impairment, are those who drop out after referral, who refuse referral or who are not referred in the first place.

### Interpersonal level

#### Information provision LVS

Information provision came forward as a facilitator as well as a barrier from both the professionals’ and patients’ perspectives. Half of the patients reported that they have not been informed by their provider about LVS or to have initiated their referral. In contrast, almost all ophthalmologists and low vision optometrists (those with the authorization to refer patients) reported to regularly inform patients about LVS. Several professionals reported to regularly hear from patients that they wished they had been informed and referred earlier, immediately at the time of diagnosis or shortly after. Although the wish for earlier information provision was also stated by several patients, most patients felt that they were referred at the right time in due course, often as a result of self-advocacy.

“*If the diagnosis is determined, they should give you options. The ophthalmologist could have told me first ‘We will first await the treatment effect and then we will see if we can possibly refer you to the LVS organization’, or something like that. That would have been much better. It would also have given me more peace of mind if someone would have said that (Female, aged 55, macular degeneration).”*

Patients who have a check-up appointment with their ophthalmologist only once in a while (e.g. every 6 months to every year) are at risk of not being informed, according to some professionals. Besides that, professionals mentioned that ophthalmologists may not pay attention to potential LVS needs of patients during every consult, because of prioritizing the medical aspect of the disease within the time constraints of consultation. In addition, ophthalmologists might not report about informing their patients, because of unclear assumptions about what LVS has to offer. For example, in young patients referrers may assume that patients are too young to have LVS needs, whereas in older patients they may assume that patients are too old to benefit from LVS. Another reason mentioned by several professionals was that ophthalmologists may predominantly use the visual acuity criterion of > 0.5 logMAR, despite the before mentioned possibilities for broader interpretation of the guideline.

In addition, several professionals stated that many patients with VI have near vision impairment as well, which is not measured in the ophthalmic practice/hospitals. As a consequence ophthalmologists may forget to refer these patients, as they relate to distance visual acuity when referring, although these patients might benefit from LVS too:

*“(…) People with wet macular degeneration who receive injections, the distance vision is often good enough and they cannot read anyway, but often we do notice that the reading acuity is bad. That group is also often overlooked and therefore not referred because of their distance vision not being bad enough. (LVS professional, female, aged 45).”*

#### Communication skills/strategies healthcare professionals

Communication skills and strategies of professionals in eye care were identified as facilitators and barriers from both perspectives. Several professionals reported to use different communication skills when discussing LVS. Listening, probing questions about daily life activities and showing empathy were mentioned to be important to investigate patients’ LVS needs.

*“I use my assessment and my communication skills to point that out, (…) a bit of sensing what people can and cannot do and whether they live alone or whether there is help, how the groceries are going, whether they need help with that, things like that (…). Well, reading, how that goes, watching TV, mobility, I think those are the most important three to always ask (Low vision optometrist, female, aged 45).”*

Some of these communication skills were also mentioned by patients to be important to investigate LVS needs or were the reason for them to think positively about LVS, for example being asked questions about daily life activities and being treated respectfully.

*“They take the time for you. That is important, the main thing is that you are a person and that people see that. (…) At the LVS that man asked me ‘When you look at me, what parts do you see of my face?’ And I started laughing (…) and I said ‘No one had ever asked me such a specific question’ (Male, aged 60, glaucoma and macular degeneration).”*

According to one patient, lacking communication skills hindered referral as there is less openness to discuss patients’ LVS needs:

*“(…) I have experience with two doctors. I think both doctors are very capable, with one doctor I talk very easily and he listens and the other ophthalmologist stays focused on the screen and it is much more difficult to have conversations with him. Then it is much more difficult to get it started (Female, aged 88, macular degeneration).”*

Both professionals and patients identified referral facilitators related to communication, including sensing the right moment to talk about LVS and repeating LVS information over time as patients might not be ready at first. In addition, managing expectations of patients about the disease prognosis and about possible future LVS needs was mentioned as another facilitator. Some professionals noted the need to motivate and encourage patients if they are hesitant about the referral by using clear examples of LVS possibilities and to illustrate why it may be useful and what to expect. This may reduce fears and may create readiness.

#### Social support

Half of the professionals stated that family or significant others play an important role during consultations. Family and significant others help to identify and specify patients’ LVS needs when patients find it difficult to express their needs.

*“And it also depends on the people that accompany patients. Sometimes there are children who are very active and who want everything to be done (for their parent). But when there is no company, or a neighbor or something like that, then of course the connection is much weaker and then they don’t care what is done. So if the patients’ company is stimulating, then it is of course much better (Ophthalmologist, female, aged 43).”*

Some patients said that family facilitated their LVS entry, by contacting and helping them register at the LVS and arranging the initial appointment. Furthermore, some professionals and patients stated that patients may be informed by friends, neighbors or peers, which can in turn initiate LVS referral.

#### Length of patient-provider relationship

The length of the patient-provider relationship was mentioned to be both a barrier and facilitator where short patient-provider relationships may hamper access to LVS, long term relationships were a facilitator. Some of the professionals working in non-academic hospitals reported having long patient-provider relationships with patients with chronic eye disease, as they see them regularly for check-up appointments. Consequently, this facilitates the opportunity to talk about LVS. Some patients also mentioned a long patient-provider relationship to stimulate LVS access, because of trust and openness to talk about LVS needs.

“Yeah, I've been treated there regularly, so of course, I've known him (rehabilitation physician) for years. To him I dared to say ‘Can't you refer me there?’ (Female, aged 74, macular degeneration).”

#### Other sources of LVS information

Patients may be informed by patient associations or other healthcare professionals (e.g. nurse, ophthalmic assistant) about LVS. Access to the internet as well as patient resources were identified as sources of information by professionals and patients. These may stimulate patients to contact LVS themselves or discuss it with their medical specialist.

### Organizational level

#### Communication between health professionals

A lack of communication between health professionals was mentioned to be a barrier for referral by some professionals. For example, ophthalmic assistants who talk with patients about their LVS needs, but subsequently forget to pass on this information to the ophthalmologists. Furthermore, some professionals mentioned not receiving communication or feedback from the LVS about their referral and patient outcomes, as this communication would provide relevant information about the effect, which they in turn could use to inform patients and would help to enhance awareness.

*“Then you get feedback about what you can do for the patient and I think if I would get more feedback about the patients I referred (…) then I think that the next referral is a bit more active because I can also include that in the conversation with the next patient who may be in doubt (Ophthalmologist, female, aged 47).”*

#### Cooperation between providers

Some professionals reported that investing in good and long collaboration between the different healthcare providers involved in the delivery of LVS promotes referral. This ongoing relationship generates efficient lines of communication, stimulates trust between professionals and facilitates referral pathways.

#### Care coordination

Lack of coordinated care was mentioned to be another barrier in the access by some professionals. They reported, that especially in treatment with anti-VEGF injections, patients tend to see different professionals for their treatment whereas no one takes responsibility for potential needs that could be met by LVS.

“*That are the people who, if you don’t watch out, are being overlooked and therefore are not referred, because both patient and doctor are busy with saving what can be saved (regarding eyesight) and then sometimes they forget that in the meantime help is also needed at another level. Especially because this group doesn’t often see their own ophthalmologist, as one day this person performs the injection and tomorrow that person, and so on (Ophthalmologist, male, aged 64).*”

In addition, some professionals said that it is not always recorded when LVS is discussed with a patient. Ophthalmologists may not know whether patients were referred or may forget to come back to it at a later stage.

#### Low vision optometric service

According to several professionals, the presence of a low vision optometric service at hospital ophthalmology departments facilitates LVS referral. Ophthalmologists sometimes may prefer to first refer to the optometrist for optical low vision aids (which may be an outpatient service as well) instead of referring directly to an outpatient LVS. According to some professionals, a low vision optometric service may also break down barriers for some patients to make use of LVS.

Optimizing remaining vision with optical low vision aids is the main reason for patients to first go to the optometric service, before considering LVS, according to some professionals. Low vision optometrists have an important signaling function and may also refer to LVS.

In some Dutch hospitals a delegation arrangement between ophthalmologists and optometrists is introduced, whereby optometrists are authorized to refer patients as well as ophthalmologists. With this arrangement, optometrists do not have to consult the ophthalmologists before referring a patient. According to some professionals, this facilitates referral as it saves time for ophthalmologists and speeds up the delivery to LVS.

#### Time constraints ophthalmic practice

According to some professionals and patients, lack of time in the ophthalmic practice forms a barrier in patients’ referral and leads to limited information provision:

“I also think that we could refer more, but we somehow don't do that, also because of the busy consultation hours (…) (Ophthalmologist, female, aged 43).”

### Community level

#### Fear of stigma

Some professionals reported that fear of stigma can be a reason for some patients to refuse LVS, e.g. low vision aids. The fear of being stereotyped and being ashamed of using low vision aids may hinder their referral.

“*Then it has a stigmatizing effect and then people say ‘I am not ready for that yet’. I always compare it with my parents who didn’t want to walk with a walker after hip rehabilitation. Then everybody would have seen that they had something. (Ophthalmologist, male, aged 42).”*

#### Distance to LVS/Transportation

Distance to LVS and problems with transportation was identified as another barrier by several professionals. Examples that were mentioned are people living remotely (e.g. on an island), elderly patients who are dependent on others and patients not wanting to be a burden for their family regarding transportation to the LVS. Some patients said that distance to LVS and transportation possibilities are aspects they considered for LVS utilization. However, all patients reported not to have problems with distance or transportation, because they either lived close to an LVS, had family who could bring them, or received taxi support by their health insurance.

#### Education of healthcare providers of LVS

Some professionals stated that education about LVS for eye care professionals involved in the delivery of LVS is important to create awareness, which in turn facilitates referral.

“Ophthalmologists have relatively little time, so we try to make the ophthalmologists, the optometrists, the technical ophthalmic assistants (…) as aware as possible about our work (LVS professional, female, aged 45).”

### Public-policy level

#### Dutch healthcare system

From both the patients’ and the professionals’ perspective the Dutch healthcare system hampers and promotes LVS access. As LVS are paid by health insurance in the Dutch healthcare system, there were no patients who experienced a barrier in the access due to costs.

“*I also have a reasonable pension, so we can manage just fine financially. (…) It (care of LVS organization) is still 100% reimbursed. (…) I have visual problems and my own compulsory deductible goes to that (Male, aged 78, macular degeneration).”*

However, according to one professional the compulsory deductible payment within the Dutch health insurance can be a barrier for some patients to purchase low vision aids. However, another professional mentioned that older patients often have comorbid diseases. For those patients, the compulsory deductible payment is often already paid for other medical care and therefore does not form a barrier. Moreover, according to one professional, restructuring of the Dutch healthcare system has increasingly led to shorter patient-provider relationships, which may be a reason why some patients with LVS needs may not be referred.

#### Regional service provision

Some professionals reported the regional provision of LVS in the Netherlands to be a facilitator in the access towards LVS as almost every potential patient can find help in their own living area.

#### Long waiting lists

According to some professionals, long waiting lists for LVS organizations form a barrier in the referral pathways. These occur as a result of staff shortages at the LVS organizations, which leads to long waiting lists. As a consequence, some patients drop out after referral.

## Discussion

Our study aimed to explore barriers and facilitators in the referral pathways to LVS of adults aged 50 or older in the Netherlands from both the professionals’ and patients’ perspective. We identified various barriers and facilitators on individual, interpersonal, organizational, community and public-policy level, which highlights the complex interplay of factors influencing the referral pathways to LVS. Our findings indicate that patients’ motivation for LVS, influenced by factors such as perceived impact of the VI, participation needs and attitudes, plays a significant role in the referral to LVS. At the same time, patients’ referral seems to be highly depended on adequate information provision about LVS and communication skills of professionals. Possessing self-advocacy skills and having a social support network as a patient seems to be an important facilitator as well.

Our results show that patients’ LVS referral and utilization seem to be dependent on a patients’ intrinsic motivation for LVS. Professionals reported only referring patients who indicated that they want to be referred. Several factors identified in this study seem to function as a barrier or facilitator for patients’ motivation. They comprise (lack of) acceptance of the VI, disease duration, lack of knowledge about LVS and attitudes of patients. In addition, we found that (not) having clear participation needs of patients seem to hinder or facilitate patients’ LVS motivation and thus referral. Eye care professionals should be aware however that not having participation needs may be related to patients not having a clear perception of the possibilities that LVS has to offer to improve quality of life. Vision-specific patient reported outcome measures (PROMs) may help professionals to identify factors related to patient motivation to guide their referral procedures [48, 49].

Our results indicate that patients who have self-advocacy skills are more likely to be referred, as they are more likely to discuss their LVS needs with their provider or contact LVS themselves. This corresponds with previous literature on LVS delivery [25, 50] and implies that patients in need of LVS, but who lack self-advocacy skills are especially at risk of not being referred to LVS. Research in other patient populations suggests that self-advocacy is a teachable skill that contributes to an individualized care trajectory that fits patients’ needs, preferences and values [51, 52]. Furthermore, communication aids for patients in medical consultations, for example Question Prompt Lists, may help patients to express their needs and enhance patient participation and information provision [53].

We found that information provision by eye care providers influences LVS referral, and has also been reported by others [31, 54, 55]. There seems to be a discrepancy between the professionals’ and patients’ experiences. Whereas almost all ophthalmologists in this study seemed to regularly inform patients about LVS and to refer them, some patients reported to have been informed late or elsewhere. Ophthalmologists’ lack of attention towards possible LVS needs, inadequate assumptions about certain subgroups not having needs (e.g. younger age groups), and lack of time were identified as important barriers. Previous literature reported delayed referral as a result of lacking information provision, which was not confirmed by our study, as most patients felt that they self-advocated or had been referred at the right time. Furthermore, improvement of procedures may be facilitated by tools, for example, electronic health record-based clinical decision support systems to stimulate timely referral and to help diminish inadequate assumptions about whom to refer [56].

The communication skills and strategy of providers seem to be important in the referral pathways as well. Lack of effective communication has been reported before as a barrier to LVS [54]. Our study showed specific communication skills that facilitate referral, such as sensing and timing the right moment to talk about LVS to patients, actively asking patients questions about daily life functioning, using clear examples, motivating patients, managing expectations and repeating information are facilitating communication strategies for discussing patients’ LVS needs and referral. These skills and strategies are in line with effective patient-provider communication and a patient-centered approach [57, 58] which may lead to increased access to care.

Social support networks of patients seemed to be a relevant facilitator as well, which is in accordance with previous work [22]. Family and other significant others present during consultations with the ophthalmologist or low vision optometrist seemed to increase needs identification. Furthermore, social support facilitated access to LVS. This implies that professionals should encourage patients to take a trusted person to consultations.

### Strengths and limitations

A strength of this study is the inclusion of both the patients’ and the professionals’ perspectives. We thereby triangulated [59] our findings, which we believe contributed to a more comprehensive understanding of factors influencing the referral pathways to LVS. Moreover, by means of member checking we established credibility. In addition, we tried to include a heterogeneous population of professionals and patients, which enhanced transferability of our results. Furthermore, we nearly met our approximated sample size per subgroup, which was found to be sufficient in terms of information power [41] during the research process for the exploratory aim of the study.

Despite our efforts, a limitation of our study is that we were only able to include patients who followed up on the referral. As a consequence, the perspective of patients who potentially would have benefited from LVS, but who remained ‘under the radar’, for example because they were not offered a referral, refused referral or did not have access due to other barriers in the referral pathway, are missing. Nonetheless, we were able to shed some light on this patient group and their related barriers and facilitators with the professionals’ point of view.

In general, a limitation of interview studies is their retrospective nature. For patients, we tried to mitigate recall bias by inviting patients who were referred to LVS not longer than 6 months ago. However, it turned out to be difficult to meet this criterion, partly caused by the corona pandemic. The initial protocol was to include patients who were referred within 6 months. Since the corona pandemic made this impossible and our funds were limited, we decided to accept also patients as participants who had been referred between 8 months and 6 years ago. This might have aggravated recall bias.

Lastly, our results relate to LVS referrals in the Dutch context, which should be taken into account when applying the results to other countries, as referral practices and LVS provision vary internationally.

## Conclusion

Our findings imply that providers’ lack of information provision, especially to patients who lack self-advocacy skills, hamper referral to LVS. At the same time, in the Dutch context, not all patients who are potentially eligible for LVS seem to want to be referred and to have rehabilitation needs. Providers should have attention for patients’ LVS needs and actively inform them and their social network about LVS to facilitate access. Educating and training providers about how and when to address LVS may help to reduce barriers in the referral pathways. In addition, referral procedures may benefit from tools that make providers more aware of LVS, e.g. referral alert tools or PROMs. The results of this study may provide eye care professionals and policy makers with insights to mitigate barriers in LVS referral procedures and to organize them more efficiently. This in turn will help to facilitate timely referral of patients who qualify, and who are ready for LVS to help them to enhance their QoL.

## Data Availability

The data generated and analyzed during the current study are not publicly available as they could compromise the privacy of the participants. The data are available from the corresponding author at reasonable request. The interview guide used to generate data is also available upon request from the corresponding author.

## Abbreviations

LVS: Low Vision Services
SD: Standard deviation
VI: Visual impairment

## References

[1] Kempen GI, Ballemans J, Ranchor AV, van Rens GH, Zijlstra GA (2012). The impact of low vision on activities of daily living, symptoms of depression, feelings of anxiety and social support in community-living older adults seeking vision rehabilitation services. Qual Life Res.

[2] Lamoureux EL, Fenwick E, Moore K, Klaic M, Borschmann K, Hill K (2009). Impact of the severity of distance and near-vision impairment on depression and vision-specific quality of life in older people living in residential care. Invest Ophthalmol Vis Sci.

[3] Rees G, Fenwick EK, Keeffe JE, Mellor D, Lamoureux EL (2009). Detection of depression in patients with low vision. Optom Vis Sci.

[4] van der Aa HPA, Comijs HC, Penninx BWJH, van Rens GHMB, van Nispen RMA (2015). Major Depressive and Anxiety Disorders in Visually Impaired Older Adults. Invest Ophthalmol Vis Sci.

[5] Schakel W, Bode C, van de Ven PM, van der Aa HPA, Hulshof CTJ, van Rens GHMB (2019). Understanding fatigue in adults with visual impairment: a path analysis study of sociodemographic, psychological and health-related factors. PLoS ONE.

[6] Hong T, Mitchell P, Burlutsky G, Samarawickrama C, Wang JJ (2014). Visual impairment and the incidence of falls and fractures among older people: longitudinal findings from the Blue Mountains Eye Study. Invest Ophthalmol Vis Sci.

[7] World Health Organisation. International statistical classification of diseases (ICD), 11th Revision, 2022 version. Available from: https://icd.who.int/browse11/l-m/en.

[8] Bourne R, Steinmetz JD, Flaxman S, Briant PS, Taylor HR, Resnikoff S (2021). Trends in prevalence of blindness and distance and near vision impairment over 30 years: an analysis for the Global Burden of Disease Study. Lancet Glob Health.

[9] Owsley C, McGwin G, Lee PP, Wasserman N, Searcey K (2009). Characteristics of Low-Vision Rehabilitation Services in the United States. Arch Ophthalmol.

[10] Luu W, Kalloniatis M, Bartley E, Tu M, Dillon L, Zangerl B (2020). A holistic model of low vision care for improving vision-related quality of life. Clin Exp Optom.

[11] Markowitz SN (2006). Principles of modern low vision rehabilitation. Can J Ophthalmol.

[12] Lim YE, Vukicevic M, Koklanis K, Boyle J (2014). Low Vision Services in the Asia-Pacific Region: Models of Low Vision Service Delivery and Barriers to Access. J Vis Impair Blind.

[13] van Nispen RMA, Virgili G, Hoeben M, Langelaan M, Klevering J, Keunen JEE (2020). Low vision rehabilitation for better quality of life in visually impaired adults. Cochrane Database Syst Rev..

[14] American Optometric Association. Clinical Practice Guidelines. n.d. [November 8, 2022]. Available from: https://www.aoa.org/practice/clinical-guidelines/clinical-practice-guidelines?sso=y.

[15] Sinclair A, Ryan B. Low vision: The essential guide for ophthalmologists The Guide Dogs for the Blind Association; 2008. Available from: https://www.rcophth.ac.uk/resources-listing/low-vision-the-essential-guide-for-ophthalmologists/.

[16] Low Vision Academy. Guidelines for the visual rehabilitation of the visually impaired 2016 [November 8, 2022]. Available from: https://en.lowvisionacademy.org/2014/09/16/guidelines-for-the-visual-rehabilitation/.

[17] Nederlands Oogheelkundig Gezelschap. Visuele beperkingen - verwijzing en revalidatie [Vision disorders: referral and rehabilitation]. 2020. Available from: https://richtlijnendatabase.nl/richtlijn/visuele_beperkingen_-_verwijzing_en_revalidatie/verwijzing_voor_revalidatie_bij_visuele_beperkingen.html.

[18] Pollard TL, Simpson JA, Lamoureux EL, Keeffe JE (2003). Barriers to accessing low vision services. Ophthalmic Physiol Opt.

[19] Khan SA, Shamanna B, Nuthethi R (2005). Perceived barriers to the provision of low vision services among ophthalmologists in India. Indian J Ophthalmol.

[20] Overbury O, Wittich W (2011). Barriers to low vision rehabilitation: the Montreal Barriers Study. Invest Ophthalmol Vis Sci.

[21] Stolwijk ML, van Nispen RMA, Verburg IWM, van Gerwen L, van Rens GHMB (2022). Trends in low vision service utilisation: A retrospective study based on general population healthcare claims. Ophthalmic Physiol Opt.

[22] Lam N, Leat SJ (2015). Reprint of: Barriers to accessing low-vision care: the patient's perspective. Can J Ophthalmol.

[23] Lam N, Leat SJ, Leung A (2015). Low-vision service provision by optometrists: a Canadian nationwide survey. Optom Vis Sci.

[24] Kaleem MA, West SK, Im L, Swenor BK (2018). Referral to Low Vision Services for Glaucoma Patients: Referral Criteria and Barriers. J Glaucoma.

[25] Kaldenberg J (2019). Low vision rehabilitation services: Perceived barriers and facilitators to access for older adults with visual impairment. Br J Occup Ther..

[26] Kaleem MA, West SK, Im LT, Swenor BK (2017). Referral to Low Vision Services for Glaucoma Patients: Referral Patterns and Characteristics of Those Who Refer. J Glaucoma.

[27] Kumar H, Monira S, Rao A (2016). Causes of Missed Referrals to Low-Vision Rehabilitation Services: Causes in a Tertiary Eye Care Setting. Semin Ophthalmol.

[28] Goldstein JE, Guo X, Boland MV, Swenor BK. Low Vision Care - Out of Site. Out of Mind. Ophthalmic Epidemiol. 2020;27(4):252–8. 10.1080/09286586.2020.1717546 31985303

[29] Sivakumar P, Vedachalam R, Kannusamy V, Odayappan A, Venkatesh R, Dhoble P (2020). Barriers in utilisation of low vision assistive products. Eye (Lond).

[30] Matti AI, Pesudovs K, Daly A, Brown M, Chen CS (2011). Access to low-vision rehabilitation services: barriers and enablers. Clin Exp Optom..

[31] O'Connor PM, Mu LC, Keeffe JE (2008). Access and utilization of a new low-vision rehabilitation service. Clin Exp Ophthalmol.

[32] Khimani K, Redmon C, Malaya L, Zaidi A, Schmitz-Brown M, Tzeng H-M (2021). Barriers to low vision care rehabilitation services for visually impaired patients in a multidisciplinary ophthalmology outpatient practice. Invest Ophthalmol Vis Sci..

[33] Sarika G, Venugopal D, Sailaja MVS, Evangeline S, Krishna KR (2019). Barriers and enablers to low vision care services in a tertiary eye care hospital: A mixed method study. Indian J Ophthalmol.

[34] Oviedo-Cáceres MdP (2022). Arias-Valencia S, Hernández-Quirama A, Ruiz-Rodríguez M, Guisasola-Valencia L. Intersectionality and access to visual rehabilitation services: Experiences of people with low vision, a qualitative study. Br J Vis Impair..

[35] Kyeremeh S, Mashige KP (2021). Availability of low vision services and barriers to their provision and uptake in Ghana: practitioners' perspectives. Afr Health Sci.

[36] Flaxman SR, Bourne RRA, Resnikoff S, Ackland P, Braithwaite T, Cicinelli MV (2017). Global causes of blindness and distance vision impairment 1990–2020: a systematic review and meta-analysis. Lancet Glob Health.

[37] Tong A, Sainsbury P, Craig J (2007). Consolidated criteria for reporting qualitative research (COREQ): a 32-item checklist for interviews and focus groups. Int J Qual Health Care.

[38] Kroneman M, Boerma W, van den Berg M, Groenewegen P, de Jong J, van Ginneken E (2016). Netherlands: Health System Review. Health Syst Transit.

[39] Green J, Thorogood N. Qualitative methods for health research. 4 ed: sage; 2018.

[40] Vasileiou K, Barnett J, Thorpe S, Young T (2018). Characterising and justifying sample size sufficiency in interview-based studies: systematic analysis of qualitative health research over a 15-year period. BMC Med Res Methodol.

[41] Malterud K, Siersma VD, Guassora AD (2016). Sample Size in Qualitative Interview Studies: Guided by Information Power. Qual Health Res.

[42] McLeroy KR, Bibeau D, Steckler A, Glanz K (1988). An ecological perspective on health promotion programs. Health Educ Q.

[43] Richard L, Potvin L, Kishchuk N, Prlic H, Green LW (1996). Assessment of the integration of the ecological approach in health promotion programs. Am J Health Promot.

[44] Birt L, Scott S, Cavers D, Campbell C, Walter F (2016). Member Checking: A Tool to Enhance Trustworthiness or Merely a Nod to Validation?. Qual Health Res.

[45] Ritchie J, Spencer L. Qualitative data analysis for applied policy research. In: Bryman A, Burgess R, editors. In: Analyzing qualitative data: Routledge; 1994. p. 173–94.

[46] Parkinson S, Eatough V, Holmes J, Stapley E, Midgley N (2016). Framework analysis: a worked example of a study exploring young people’s experiences of depression. Qual Res Psychol.

[47] Midgley N, Parkinson S, Holmes J, Stapley E, Eatough V, Target M (2015). Beyond a diagnosis: The experience of depression among clinically-referred adolescents. J Adolesc.

[48] Rausch-Koster TP, Luijten MAJ, Verbraak FD, van Rens GHMB, van Nispen RMA (2022). Calibration of the Dutch EyeQ to Measure Vision Related Quality of Life in Patients With Exudative Retinal Diseases. Transl Vis Sci Technol..

[49] Fenwick EK, Barnard J, Gan A, Loe BS, Khadka J, Pesudovs K (2020). Computerized Adaptive Tests: Efficient and Precise Assessment of the Patient-Centered Impact of Diabetic Retinopathy. Transl Vis Sci Technol..

[50] Burton AE, Gibson JM, Shaw RL (2016). How do older people with sight loss manage their general health? A qualitative study. Disabil Rehabil.

[51] Schmidt EK, Faieta J, Tanner K (2020). Scoping Review of Self-Advocacy Education Interventions to Improve Care. OTJR Occup Particip Health.

[52] Alsbrook KE, Donovan HS, Wesmiller SW, Hagan TT (2022). Oncology Nurses' Role in Promoting Patient Self-Advocacy. Clin J Oncol Nurs.

[53] Sansoni JE, Grootemaat P, Duncan C (2015). Question Prompt Lists in health consultations: A review. Patient Educ Couns.

[54] Southall K, Wittich W (2012). Barriers to Low Vision Rehabilitation: A Qualitative Approach. J Vis Impair Blind.

[55] Wittich W, Canuto A, Overbury O (2013). Overcoming barriers to low-vision rehabilitation services: improving the continuum of care. Can J Ophthalmol.

[56] Guo X, Swenor BK, Smith K, Boland MV, Goldstein JE (2021). Developing an Ophthalmology Clinical Decision Support System to Identify Patients for Low Vision Rehabilitation. Transl Vis Sci Technol..

[57] Makoul G, van Dulmen S. What Is Effective Doctor–Patient Communication? Review of the Evidence. In: Clinical Communication in Medicine. Hoboken: Wiley; 2015. p.30–9. Available from: 10.1002/9781118728130.ch5. [cited 2022 Aug 12] .

[58] Illingworth R. Patient-Centredness. In: Clinical Communication in Medicine [Internet]. Hoboken: Wiley; 2015. p.40–8. Available from: 10.1002/9781118728130.ch6. [cited 2022 Aug 12]

[59] Farmer T, Robinson K, Elliott SJ, Eyles J (2006). Developing and Implementing a Triangulation Protocol for Qualitative Health Research. Qual Health Res.

